# Recent advances in osteoarthritis research: A review of treatment strategies, mechanistic insights, and acupuncture

**DOI:** 10.1097/MD.0000000000041335

**Published:** 2025-01-24

**Authors:** Zhengchi Lou, Fan Bu

**Affiliations:** a The Third Affiliated Hospital of Xinxiang Medical University, Henan, China.

**Keywords:** osteoarthritis, pathological mechanism, research advancement, treatment strategies

## Abstract

Osteoarthritis (OA) is a chronic joint condition affecting millions worldwide, characterized by the gradual degeneration of joint cartilage, leading to pain, stiffness, and functional impairment. Although the pathogenesis of OA is not fully understood, the roles of inflammation, metabolic dysregulation, and biomechanical stress are increasingly recognized. Current treatments, including pharmacotherapy, physical therapy, and surgical interventions, aim to alleviate symptoms and improve quality of life, yet they face limitations and challenges. In recent years, researchers have explored a variety of new treatment strategies, such as molecular targeted therapy, biologic treatments, regenerative medicine, and lifestyle modifications, aiming to directly address the root causes and complex mechanisms of OA. This review aims to summarize the latest research advancements to provide fresh perspectives for clinical treatment and lay the foundation for future research and development of treatment strategies for OA.

## 1. Introduction

Currently, the treatment of osteoarthritis (OA) primarily focuses on symptom management, aiming to alleviate pain, improve joint function, and enhance patients’ quality of life.^[1]^. However, these approaches, including nonsteroidal anti-inflammatory drugs (NSAIDs), analgesics,^[2]^ physical therapy, and joint replacement surgery,^[3]^ are largely palliative and fail to effectively halt or reverse disease progression. This symptom-centric strategy reflects the limited understanding of the complex pathophysiology of OA, which has hindered the development of targeted therapies.

Research has revealed that OA pathogenesis involves multiple pathological processes, such as cartilage degeneration, subchondral bone remodeling, synovial inflammation, and metabolic dysregulation.^[4]^ These pathological changes often manifest in the early stages of the disease, yet existing treatment options generally do not address these underlying mechanisms. Notably, there is a significant lack of disease-modifying osteoarthritis drugs (DMOADs) capable of altering the natural course of OA to slow or prevent its progression.^[5]^

This therapeutic gap carries profound implications. With the increasing prevalence of OA driven by global aging populations and the obesity epidemic, the disease is causing substantial declines in patient quality of life while placing a tremendous economic burden on healthcare systems. Therefore, the development of DMOADs targeting the core pathological processes of OA is not only critical for alleviating patient suffering but also essential for reducing the socioeconomic impact of the disease.

The focus of DMOAD development should include slowing cartilage degeneration, modulating synovial inflammation, mitigating metabolic abnormalities, and promoting joint regeneration. The successful development of such therapies has the potential to offer groundbreaking treatment options for OA patients and significantly transform the management of this debilitating condition.

## 2. Epidemiology

OA, the most common type of arthritis, affects approximately 350 million people worldwide, making up 15% of the population.^[6]^ The Global Burden of Disease Study 2019^[7]^ revealed a dramatic 113.25% increase in global OA cases from 1990 to 2019, from 247.51 to 527.81 million. Notably, in 2019, China, India, and the United States reported the highest numbers of OA cases, with OA’s prevalence continuing to rise annually, signaling a growing degenerative threat to global health and healthcare systems.

Looking ahead to 2040, projections^[8]^ based on current trends suggest a 49% increase in diagnosed U.S. adults, reaching 78.4 million, and a 52% rise in those experiencing arthritis-related limitations, totaling 34.6 million. This anticipated growth underscores the urgent need for targeted interventions and research focused on understanding OA’s multifaceted impacts.

## 3. Risk factors

### 3.1. Joint shape

Joint shape serves as a risk factor for OA through its influence on the biomechanical environment of the joint.^[9]^ Variations in joint shape, such as abnormal femoral head-neck junction (cam morphology), acetabular dysplasia, or deviations in the angles of the femur and tibia, can lead to altered load distribution and increased mechanical stress on the articular cartilage.^[10]^ This abnormal stress can accelerate cartilage wear and degeneration, predisposing the joint to OA. Previous studies have linked variations in bone/joint shape with OA at the hip and knee, with recent research clarifying the relationship between these joints. A study^[11]^ using data from the Johnston County Osteoarthritis Project found that hips with moderate radiographic OA were more prone to cam morphology in both genders, and protrusio acetabuli in females. The Rotterdam Study^[12]^ identified cam deformity and acetabular dysplasia as independent predictors for hip OA. The Osteoarthritis Initiative data^[13]^ revealed gender differences in the mediation effects for knee OA, with specific proximal tibiofemoral joint morphological features linked to OA onset. Genetic epidemiology review^[14]^ suggests specific genes are related to joint shape and OA. Pathological developmental abnormalities of the hip such as DDH, Perthes’ disease, and SCFE are known to lead to osteoarthritis and hip replacement. Recent findings highlight that subtle joint shape variations also predispose to degenerative changes, typically assessed via clinical X-rays.

### 3.2. Age

Age is a significant risk factor for the progression of OA, markedly influencing its onset and severity.^[15]^ As individuals age, the wear and tear on joint cartilage accelerates, elevating the likelihood of developing OA. This degenerative process results in inflammation, pain, stiffness, and reduced mobility in the joints, thereby adversely affecting quality of life. The prevalence of OA is notably higher among the elderly, particularly in weight-bearing joints like the knees and hips, making age a critical driver in the disease’s development.^[16]^ In older populations, OA often coexists with other chronic conditions, potentially exacerbating the adverse outcomes associated with these diseases.^[17]^

### 3.3. Obesity

Obesity is a significant risk factor for OA, exacerbating the condition through several mechanisms.^[18]^ Firstly, obesity increases mechanical stress on joints, particularly in the lower limbs such as the knees, accelerating the wear and tear of joint cartilage.^[19]^ Secondly, obesity is associated with a systemic inflammatory state, with adipose tissue releasing inflammatory mediators like TNF-α and IL-6, which directly damage joint cartilage.^[20]^ Additionally, obesity is linked to metabolic abnormalities that further worsen OA through promoting inflammation and affecting cellular signaling within joint cartilage.^[21]^ Finally, obesity can lead to biomechanical changes, such as alterations in gait and posture, increasing stress on specific joints.^[22]^ Therefore, obesity significantly increases the risk of OA by enhancing mechanical load, fostering inflammation, inducing metabolic abnormalities, and causing biomechanical changes.

### 3.4. Gender

Elderly individuals are particularly vulnerable to OA, with women being more affected than men, a disparity that extends across various joints. This gender disparity is further illustrated by the distribution among adult arthritis patients, where 59.3% are female. Additionally, age-standardized prevalence rates are higher in women (24.2%) compared to men (18.5%).^[23]^ Research, including a study^[24]^ from Catalonia’s Spanish SIDIAP database, underscores this gender disparity, showing a significant risk increase in hip and knee OA among women aged 50 to 75. Unlike men, who experience a continuous rise in OA across all joints with age, women’s risk for hand OA notably spikes post-menopause, aligning with knee OA prevalence in the elderly.

### 3.5. Race

Significant racial and ethnic differences in OA incidence are also evident in the United States.^[25]^ African Americans face a higher prevalence and severity of lower extremity OA than Caucasians,^[26]^ though the opposite trend is observed in hand OA within the Osteoarthritis Initiative cohort.^[27]^ Interestingly, Chinese women have a 45% higher incidence of knee OA than Caucasian women, highlighting diverse OA impacts across different populations. In a study^[28]^ aimed at characterizing the radiographic progression of knee osteoarthritis (KOA) across different races and genders over four years, the findings revealed that African-American males at risk for KOA had a higher risk of medial joint space narrowing compared to white females and males. Notably, African-American males showed greater annual medial joint space width loss. The study suggests that established risk factors significantly contribute to the racial and gender disparities in KOA progression rates.

## 4. Type

OA is categorized into 2 main types: primary and secondary,^[29]^ each with distinct characteristics and causes. Primary OA is the most common form, whose exact causes are not fully understood but are broadly attributed to aging, genetic predisposition, metabolic disorders, and biomechanical changes.^[30]^ This type of OA predominantly affects weight-bearing and frequently used joints, such as the knees, hips, hands, and spine, especially common among the elderly. It is characterized by the gradual degeneration of joint cartilage, leading to pain, functional impairment, and a diminished quality of life. Over time, it may result in significant joint damage and deformity.^[31]^

Secondary OA arises from specific external factors or internal diseases, including but not limited to joint injuries, chronic overuse, joint infections, inflammatory joint diseases (like rheumatoid arthritis),^[32]^ congenital joint anomalies,^[33]^ and certain metabolic diseases.^[34]^ This type of OA can occur in any joint of the body, and while its pathological changes and clinical manifestations may resemble those of primary OA, its management and treatment require addressing the underlying causes to slow disease progression and improve the patient’s quality of life.^[31]^

## 5. Pathogenesis

### 5.1. Degeneration of articular cartilage

The progressive degeneration and damage to articular cartilage constitute the primary pathological mechanism of OA, leading to the roughening of the joint surface and subsequently impairing the joint’s normal movement and function.^[35]^

Articular cartilage, found in synovial joints, is a specialized type of connective tissue that functions to provide a smooth, lubricated surface for joint movements and to efficiently transmit loads with minimal friction.^[36]^ Adult articular cartilage lacks blood vessels, nerves, or lymphatic channels, thereby possessing limited self-healing capabilities once injured. Chondrocytes, the sole cell type within articular cartilage, are essential for the maintenance of the extracellular matrix (ECM), predominantly composed of water, collagen, and proteoglycans. These components are crucial for maintaining the unique mechanical properties of articular cartilage.^[37]^ In adulthood, the regenerative and reparative capacities of articular cartilage are significantly limited due to its avascularity. With age or excessive use, the intensified degradation and abnormal differentiation of chondrocytes lead to the loss of the ECM, thus initiating the onset of OA. Chondrocytes respond sensitively to the physical properties of their surrounding environment, including the composition of the ECM and the mechanical forces applied during joint loading. These cells perceive these changes through an array of mechano-sensitive receptors and channels, activating complex downstream signaling pathways that regulate cellular activities crucial to the pathology of OA.^[38]^ In OA, the catabolic processes not only degrade the functional aspects of the ECM but also alter the composition and viscoelastic properties of the ECM produced by chondrocytes. These alterations result in an abnormal loading environment that fosters cellular dysfunction and inflammation. Articular cartilage maintains homeostasis by responding at the molecular level to various physiological stresses, including mechanical stress. Disruption of this balance can lead to the development and progression of OA.^[39]^

### 5.2. Change in subchondral bone

The subchondral bone undergoes critical transformations that play a pivotal role in the disease’s progression in OA.^[40]^ These transformations include the formation of osteophytes, an increase in bone density (sclerosis), and changes in joint morphology. Such alterations not only serve as biomechanical adaptations in response to the degeneration of articular cartilage but also signify the disease’s progression.^[41]^

The subchondral bone, situated beneath the articular cartilage, is integral to the joint structure, supporting the cartilage and participating in weight-bearing and the transmission of forces, thereby maintaining joint stability and functionality. The development of osteophytes is a reactive adaptation to increased load and the loss of cartilage protection, aimed at expanding the joint surface to mitigate stress. However, these can impede joint mobility, leading to pain and functional limitations.^[42]^ Sclerosis of the subchondral bone, reflecting its adaptive thickening under continuous load, aims to fortify the bone. Yet, it may induce pain and restrict movement. Alterations in the joint’s shape directly impact its stability and function, leading to discomfort and restricted mobility.^[43]^ Subchondral sclerosis, particularly evident in OA, denotes increased bone tissue density below the articular cartilage, a characteristic of the disease’s progression. It indicates an adaptive response to injury that could worsen cartilage degeneration by altering blood supply, thus accelerating OA’s advancement.^[44]^ This interplay between the subchondral bone and articular cartilage highlights the integrated nature of joint pathology in OA, underscoring the complexity of its progression and the multifaceted approach needed for management.

### 5.3. Synovial inflammation

Synovial inflammation is considered a critical mechanism in the OA’s progression.^[45]^ The synovium is a thin tissue lining the interior of the joint cavity, responsible for producing synovial fluid that lubricates and nourishes the joints. Synovial inflammation refers to the inflammatory process occurring within the synovial tissue of joints. It damages the articular cartilage directly through inflammatory mediators such as TNF-α and interleukins, accelerating cartilage degeneration^[46]^; concurrently, it induces the proliferation and scarring of synovial tissue, disrupting normal synovial fluid production and worsening joint lubrication, thereby impairing joint function.^[47]^ Hence, synovitis is both a symptom and a direct cause of pain and functional loss in OA, present at any stage of the disease. This inflammatory response can be triggered by various factors, including fragments from degenerated articular cartilage, mechanical stimuli, and the activation of inflammatory cells and mediators. Synovial inflammation leads to thickening of the synovial tissue, angiogenesis, and infiltration by inflammatory cells, which release cytokines and enzymes, further damaging the articular cartilage and causing joint pain and swelling.^[48]^

Histopathological analyses and Imaging studies have shown that synovitis can facilitate the pathogenesis of OA.^[49]^ The normal synovium consists of two layers. The inner layer, also known as the lining layer, mainly comprises synovial macrophages and fibroblasts, playing a crucial role in maintaining joint homeostasis. The outer layer consists of various connective tissues that assist in various joint functions.^[50]^ However, in OA synovium, there is an increased number of cells in the lining layer, especially synovial macrophages.^[51]^ In the synovial environment, pro-inflammatory cytokines secreted by M1 macrophages, such as TNF-α and IL-1β, promote chondrocyte death and matrix breakdown, accelerating cartilage degeneration and inhibiting regeneration.^[52]^

Imaging studies^[53]^ have shown that synovitis in OA is patchily distributed in different anatomical parts of the synovium, including the suprapatellar, infrapatellar, parapatellar areas, and the lateral and medial sides of the infrapatellar, as well as adjacent to the posterior cruciate ligament. A study reported a correlation between patients’ reported knee pain patterns and the locations of synovitis; specifically, pain in the suprapatellar area is highly correlated with suprapatellar synovitis demonstrated by MRI.^[54]^

### 5.4. Oxidative stress

Oxidative stress plays a crucial role in the pathogenesis of OA, characterized by an imbalance between excessive production of reactive oxygen species (ROS) and the insufficient capacity of the antioxidant defense system.^[55]^ This imbalance is prominently observed in chondrocytes and extends to affect the synovial tissue and subchondral bone ROS are primarily generated through mitochondrial oxidative phosphorylation, where electron leakage occurs, and by the enhanced activity of NOX family enzymes in inflammatory environments. Studies have shown that the expression of NOX2 and NOX4 is upregulated in OA patients, further exacerbating ROS production.^[56]^ These oxidative molecules directly damage key components of the ECM, such as collagen and proteoglycans, undermining the mechanical stability and function of the joint. Moreover, ROS regulate pro-inflammatory pathways, including NF-κB, leading to the overexpression of MMPs and ADAMTS, which accelerates ECM degradation.^[57]^

### 5.5. Translation and refinement: protease activity

Protease activity is a critical driver of cartilage degradation in OA, primarily mediated by MMPs and ADAMTS. Among these, MMP-13 and ADAMTS-5 play central roles by degrading collagen and aggrecan, respectively, which are essential for maintaining cartilage structure and elasticity.^[58]^ In OA, the activity of these proteases is markedly elevated, disrupting the ECM and accelerating cartilage degeneration.

The overexpression of MMP-13 and ADAMTS-5 is largely driven by pro-inflammatory cytokines, such as IL-1β and TNF-α, which activate signaling pathways including NF-κB and MAPK.^[59]^ Oxidative stress further amplifies these processes, creating a feedback loop that intensifies ECM breakdown. Additionally, ECM fragments released by proteolytic activity act as DAMPs, triggering further inflammation and perpetuating joint damage.^[60]^

### 5.6. Epigenetic modification

RNA activity, regulate key pathways involved in cartilage homeostasis, inflammation, and ECM remodeling. Dysregulation of epigenetic mechanisms contributes to the progression of OA by promoting chondrocyte dysfunction and enhancing pro-inflammatory and catabolic activities in joint tissues.^[61]^

DNA methylation alterations have been linked to aberrant expression of matrix-degrading enzymes, such as MMPs and ADAMTS, and inflammatory mediators like IL-1β and TNF-α.^[62]^ Hypomethylation in promoters of these genes increases their expression, exacerbating cartilage degradation and inflammation. Similarly, histone modifications, including acetylation and methylation, modulate chromatin structure and transcriptional activity of OA-related genes. HDACs, for instance, are often overactive in OA, suppressing the expression of protective genes that maintain cartilage integrity.^[63]^

Non-coding RNAs, particularly miRNAs and lncRNAs, also play a crucial role in OA progression. MiRNAs such as miR-140 and miR-146-5p regulate cartilage ECM synthesis and inflammation.^[64]^ Their dysregulation disrupts homeostatic signaling, leading to increased catabolic activity and reduced regenerative capacity. LncRNAs influence chondrocyte metabolism and inflammatory responses, further contributing to joint tissue degeneration.

**Figure 1.**
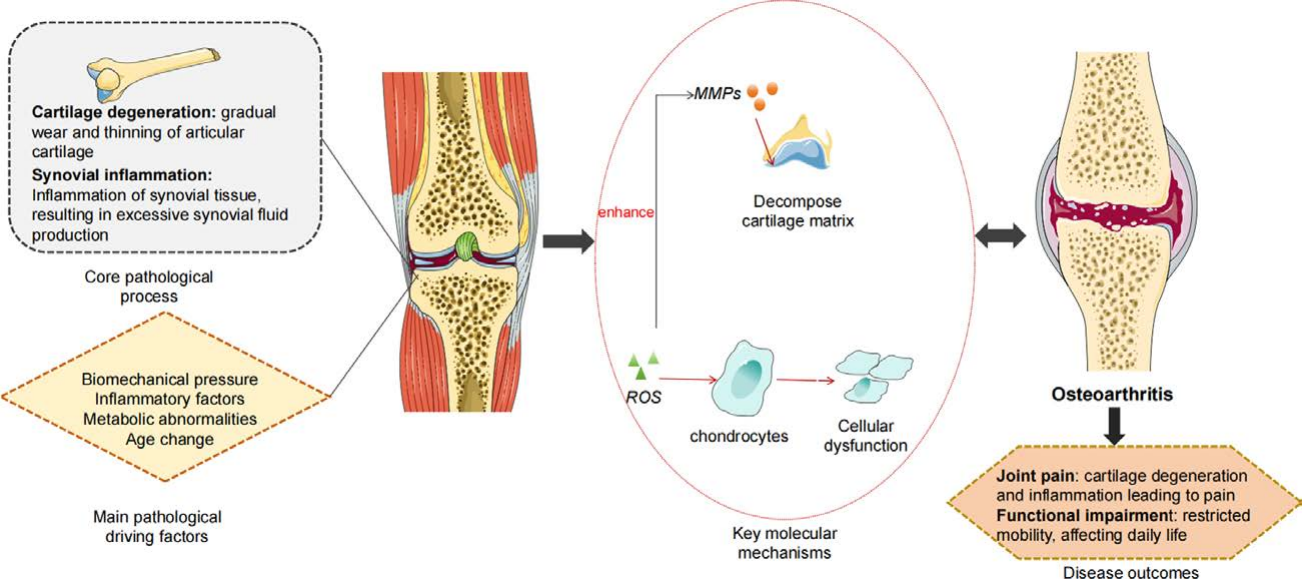
Overview of the key mechanisms in osteoarthritis (OA) pathogenesis.

This chapter elaborates on the six key mechanisms of OA. Figure 1 integrates these mechanisms, illustrating their interrelationships and dynamic changes in a visual format to further elucidate the complexity of the pathological processes in OA.

## 6. Treatment

### 6.1. NSAIDs

NSAIDs are widely used to alleviate pain, reduce inflammation and fever, especially in the treatment of inflammatory diseases such as OA. NSAIDs mainly act by inhibiting the activity of cyclooxygenase (COX) enzymes, which are crucial in the synthesis of prostaglandins, molecules that play significant roles in pain and inflammatory responses.^[65]^ Some NSAIDs, such as ibuprofen, naproxen, diclofenac, meloxicam, ketoprofen, and indomethacin, inhibit both Cox-1 and Cox-2, while others selectively inhibit Cox-2, such as etoricoxib and celecoxib.^[66]^ COX-1 is constitutively expressed in most tissues, playing a crucial role in maintaining physiological functions, such as gastrointestinal protection and blood flow regulation. In contrast, COX-2, while also inherently expressed in certain tissues, is significantly upregulated in response to inflammation and external injury, particularly at peripheral damage sites and within the central nervous system.^[67]^ The upregulation of COX-2 facilitates the production of inflammatory mediators, such as prostaglandins, prostacyclins, and thromboxanes, which are key signaling molecules regulated through the metabolism of arachidonic acid by COX enzymes.^[68]^ These mediators play vital roles during inflammation, including inducing pain, promoting inflammation, and affecting vascular functions.^[69]^

In a rat model study, Niibori M^[70]^ assessed how acetylsalicylic acid influences central sensitization associated with OA pain. Secondary hyperalgesia was measured through the Von Frey test, and the expressions of ASIC3 in dorsal root ganglia and TNF-α in the spinal cord were analyzed. Results showed that acetylsalicylic acid significantly reduced secondary hyperalgesia and decreased ASIC3 and TNF-α expressions, suggesting its potential to alleviate OA pain through mechanisms independent of COX-2 inhibition. Through an investigation in female Sprague-Dawley rats with OA, Paglia D^[71]^ explored how naproxen, a NSAID, affects the degeneration of articular cartilage. OA was induced via destabilization of the medial meniscus. OA severity and cartilage depth were assessed using histological OARSI scores and µCT. Results indicated that naproxen-treated rats showed significantly lower OARSI scores and greater cartilage depth compared to placebo, suggesting naproxen’s potential in reducing cartilage degradation in OA. The study by Wen Z^[72]^ explored the effects of etoricoxib, a COX-2 selective inhibitor, on both the progression of OA and the behaviors related to pain in rats, following anterior cruciate ligament transection. Assessed outcomes included nociceptive behavior, changes in knee joint width, cartilage histopathology, and expression of TGF-β and NGF in cartilage. Results showed that etoricoxib significantly reduced cartilage degeneration and improved nociceptive behavior compared to the control group, suggesting it may slow OA progression and modulate chondrocyte metabolism by altering the expression of NGF and TGF-β.

### 6.2. Acetaminophen

Acetaminophen, despite being one of the world’s most frequently used analgesics for treating mild to moderate OA pain, has a mechanism of action that remains somewhat contentious and not fully understood.^[73]^ Its primary mode of analgesic effect is thought to be through mild inhibition of the COX enzymes within the central nervous system, which reduces the perception of pain and decreases the production of prostaglandins, key players in the processes of pain and inflammation.^[74]^ Although acetaminophen’s anti-inflammatory properties are not as pronounced as those found in NSAIDs, it offers a notable advantage of having fewer gastrointestinal side effects, making it an ideal alternative for OA patients who need to avoid the adverse effects associated with NSAIDs.^[75]^

In an RCT study,^[76]^ researchers evaluated the efficacy and safety of taking acetaminophen extended-release (ER) tablets three times daily compared to placebo in alleviating symptoms and signs of hip or knee osteoarthritis. The results indicated that acetaminophen ER was significantly more effective than placebo in reducing physical function impairment, improving patients’ global assessment of treatment response, and alleviating pain. Additionally, acetaminophen ER showed advantages over placebo in improving stiffness and overall health scores. This study demonstrates that acetaminophen ER, as an over-the-counter medication, can effectively relieve the symptoms of hip or knee osteoarthritis and is well-tolerated. Gao J^[77]^ investigated the mechanism behind acetaminophen’s effects on inflammation and extracellular matrix degradation in human chondrocytes, a molecular process not yet fully understood. Employing the C28/I2 chondrocyte cell line and simulating an inflammatory environment with interleukin-1β, researchers explored the influence of acetaminophen and a methylation inhibitor, cycloleucine, on the modification of RNA N6-methyladenosine (m6A) and the expression of related proteins. Their findings revealed that interleukin-1β elevated m6A levels within cells, altering the balance of m6A regulatory proteins, notably by increasing methyltransferase like 3 and reducing AlkB family member 5 expression. Cycloleucine inhibited the interleukin-1β-induced inflammatory and extracellular matrix degradation signals by curtailing RNA m6A modification. Conversely, acetaminophen treatment reversed the alterations induced by interleukin-1β, mitigated the secretion of inflammatory cytokines like interleukin-6, interleukin-8, and tumor necrosis factor-α, and modulated the expression of matrix metalloproteinase-13, Collagen X, Collagen II, and aggrecan. Overexpression of AlkB family member 5 enhanced chondrocyte viability and reduced inflammation and matrix degradation, indicating acetaminophen modulated chondrocyte inflammatory responses and extracellular matrix synthesis by regulating RNA m6A levels and associated protein expressions. This shed light on acetaminophen’s analgesic action mechanism in osteoarthritis treatment.

### 6.3. Joint replacement surgery

Joint replacement surgery, also known as arthroplasty, treats OA by removing and replacing a damaged joint with an artificial one. This surgery is typically considered when OA causes severe pain, disability, and significantly impairs quality of life, and when more conservative treatments, like medication and physical therapy, have failed to provide relief.^[78]^

The goal of joint replacement is to alleviate pain, restore function, and improve the patient’s ability to perform daily activities. The most commonly replaced joints are the hips and knees, though shoulders, ankles, and other joints can also undergo replacement.

### 6.4. Acupuncture

Acupuncture, a non-pharmacological and noninvasive complementary and alternative therapy, involves inserting and rotating needles at specific points on the body to regulate Qi or life energy, while also stimulating the human nervous system.^[79]^ It is believed to reduce pain and inflammation associated with diseases such as OA. This process works by triggering the release of natural painkillers like endorphins and serotonin, as well as affecting the immune system and blood circulation. Additionally, acupuncture can improve musculoskeletal functions by inhibiting the activity of certain inflammatory cells, relaxing muscles, increasing flexibility, and enhancing joint mobility.^[80]^ These comprehensive effects not only help alleviate symptoms such as pain, stiffness, and joint swelling in OA patients but may also promote the natural healing process, slow down the progression of the disease, and have a positive impact on the treatment of various musculoskeletal disorders.

Clinical trials have confirmed acupuncture as an affordable and safe option, demonstrating its efficacy in treating OA. The pathogenesis of OA involves inflammatory and cytokine mediators, compounds, signaling pathways, genetic, and biomechanical factors that lead to cartilage degradation, with IL-1β and TNF-α playing a pivotal role.^[81]^ These cytokines activate various signaling pathways, including MAPK and TLR4,^[82]^ and promote the production of NO and PGE2,^[83]^ accelerating the degradation of collagen II and triggering OA.^[84]^ Acupuncture mitigates OA symptoms and facilitates functional recovery by suppressing the overexpression of these inflammatory factors, inhibiting signaling pathways, boosting antioxidants, and preventing the hypertrophic differentiation of chondrocytes.^[85]^ Moreover, acupuncture effectively suppresses the overexpression of IL-1β and TNF-α in cartilage, synovium, and subchondral bone, closely linked to the upregulation of MMP1, MMP2, and MMP3, leading to characteristic cartilage degradation. By downregulating the expression and secretion of IL-1β and TNF-α, acupuncture effectively controls inflammation and modulates various signaling pathways associated with OA development, such as Notch, Wnt/β-catenin, and MAPK, showcasing its potential to inhibit the OA progression.^[86]^

Tan Q^[87]^ utilizing 18F-fluorodeoxyglucose positron emission tomography and micro-computed tomography imaging, aimed to investigate whether acupuncture could inhibit inflammation and bone destruction in a rat model of KOA induced by sodium iodoacetate. The results indicated that acupuncture treatment significantly suppressed inflammation and bone destruction. Compared to the KOA group, acupuncture reduced glucose uptake, alleviated cartilage damage, synovial inflammation, and fibrosis in the infrapatellar fat pad, and after acupuncture treatment, there was a significant increase in the number, volume, and thickness of trabeculae and a decrease in the spacing between them, demonstrating that acupuncture can effectively inhibit severe bone erosion and protect the subchondral bone in KOA. Liu H^[88]^ explored the effect of SIRT1 on the efficacy of acupuncture in treating OA cartilage degeneration. The findings revealed that acupuncture effectively mitigated the upregulation of inflammatory factors TNF-α and IL-2, as well as proteins related to cartilage matrix degradation and NF-κB signaling in OA rat cartilages. Moreover, it counteracted morphological damage, SIRT1 downregulation, and the loss of proteins essential for cartilage matrix synthesis. Silencing SIRT1 reversed the beneficial impacts of acupuncture on these OA-associated alterations, except for apoptosis, which was not evaluated under SIRT1 silencing conditions. In conclusion, acupuncture delayed the progression of OA-associated cartilage degeneration by upregulating SIRT1 expression, thereby inhibiting chondrocyte apoptosis, inflammation, NF-κB signaling activation, and cartilage matrix degradation.

## 7. Comparative evaluation of treatment strategies

Emerging therapeutic strategies for osteoarthritis (OA), including molecular targeted therapies, biologics, regenerative medicine, and epigenetic modulation, are increasingly shifting focus from symptom management to disease modification. While these approaches show varying degrees of promise in altering disease progression, they also face significant limitations.

Molecular targeted therapies, such as ADAMTS-5 inhibitors and IL-1β antagonists, have demonstrated efficacy in reducing cartilage degradation in preclinical studies. However, clinical trials have revealed limited long-term joint protection, particularly in diverse patient populations. Biologic therapies, including mesenchymal stem cell (MSC) injections and growth factors, have shown positive outcomes, especially in early-stage OA. A recent phase III trial highlighted MSCs’ ability to improve knee joint function significantly, though questions remain regarding long-term efficacy and immune-related risks. Regenerative medicine approaches, such as CRISPR-Cas9 gene editing and 3D bioprinting, offer groundbreaking potential for cartilage repair but face challenges related to safety and ethical concerns. Epigenetic therapies, such as HDAC inhibitors and miRNA modulation, have shown promise in mitigating cartilage degeneration, but technical challenges like delivery efficiency and off-target effects persist.

While each of these therapies offers distinct advantages, none currently provides a comprehensive solution for modifying OA progression. Future research should prioritize integrating these strategies to maximize synergistic effects and improve long-term outcomes for patients.

## 8. Future

DirectionsFuture research should address the limitations of current OA therapies, focusing on the development of effective DMOADs. Key priorities include a deeper understanding of OA’s multifactorial mechanisms, such as oxidative stress, protease activity, and epigenetic modifications, using multi-omics approaches to identify new therapeutic targets.

Innovative strategies, including multi-target combination therapies, should be explored to overcome the limitations of single-mechanism treatments. Advances in drug delivery systems, such as nanotechnology, could enhance treatment efficacy and minimize side effects. Regenerative medicine approaches, including stem cell therapies and gene editing, require further large-scale clinical validation to confirm long-term safety and cost-effectiveness.

Additionally, personalized treatment plans based on molecular profiling hold promise for improving outcomes. Interdisciplinary and international collaborations are essential to accelerate innovation, supported by long-term patient cohorts to evaluate the real-world impact of emerging therapies. These efforts will pave the way for transforming OA management and patient care.

## 9. Conclusions

The primary goals in treating OA are to alleviate pain, improve joint function, enhance the quality of life for patients, and slow the progression of the disease as much as possible. Given the complexity and multifactorial nature of OA, devising an effective treatment plan typically requires collaboration from a multidisciplinary team. This includes an initial assessment of the patient’s symptom severity, affected joints, and overall health, while also considering the patient’s lifestyle, activity level, and preferences and responses to various treatment options. Such a comprehensive and personalized approach not only offers optimal pain management and functional improvement plans for patients but also minimizes potential side effects of treatment. This review thoroughly examines the latest advancements in OA research, highlighting innovative treatment strategies and insights into mechanisms, emphasizing the importance of a multidisciplinary method for effective management. The exploration of new treatment options, rooted in a deepening understanding of OA’s underlying mechanisms, offers hope for more targeted and effective interventions. Continuous research into the molecular pathways driving OA and the development of new therapeutic drugs is crucial for advancing our ability to combat this debilitating disease. This review encourages ongoing research and collaboration to unlock further advancements in treatment strategies.

## Author contributions

**Conceptualization:** Fan Bu.

**Funding acquisition:** Zhengchi Lou.

**Methodology:** Zhengchi Lou.

**Writing – original draft:** Zhengchi Lou.

**Writing – review & editing:** Fan Bu.
